# A Pilot Feasibility Randomized Controlled Trial on the Ontario Brain Injury Association Peer Support Program

**DOI:** 10.3390/jcm10132913

**Published:** 2021-06-29

**Authors:** Ben B. Levy, Dorothy Luong, Mark T. Bayley, Shane N. Sweet, Jennifer Voth, Monika Kastner, Michelle L. A. Nelson, Susan B. Jaglal, Nancy M. Salbach, Ruth Wilcock, Carla Thoms, John Shepherd, Sarah E. P. Munce

**Affiliations:** 1 Temerty Faculty of Medicine, University of Toronto, Toronto, ON M5S 1A8, Canada; ben.levy@mail.utoronto.ca; 2 Toronto Rehabilitation Institute, University Health Network, Toronto, ON M5G 2A2, Canada; dorothy.luong@uhn.ca (D.L.); mark.bayley@uhn.ca (M.T.B.); nancy.salbach@utoronto.ca (N.M.S.); 3 Institute of Health Policy, Management, and Evaluation, University of Toronto, Toronto, ON M5T 3M6, Canada; michelle.nelson@sinaihealthsystem.ca (M.L.A.N.); susan.jaglal@utoronto.ca (S.B.J.); 4 Rehabilitation Sciences Institute, University of Toronto, Toronto, ON M5G 1V7, Canada; john.shepherd@mail.utoronto.ca; 5 Department of Kinesiology and Physical Education, McGill University, Montreal, QC H2W 1S4, Canada; shane.sweet@mcgill.ca; 6 Centre for Interdisciplinary Research in Rehabilitation of Greater Montreal, Montreal, QC H3S 1M9, Canada; 7 Hotel Dieu Grace Healthcare, Windsor, ON N9C 3Z4, Canada; jennifer.voth@hdgh.org; 8 North York General Hospital, Toronto, ON M2K 1E1, Canada; monika.kastner@nygh.on.ca; 9 Lunenfeld-Tanenbaum Research Institute, Toronto, ON M5G 1X5, Canada; 10 Department of Physical Therapy, University of Toronto, Toronto, ON M5G 1V7, Canada; 11 Ontario Brain Injury Association, St. Catharines, ON L2V 4Y6, Canada; rwilcock@obia.on.ca (R.W.); cthoms@obia.on.ca (C.T.); 12 Department of Occupational Science and Occupational Therapy, University of Toronto, Toronto, ON M5G 1V7, Canada

**Keywords:** peer support, traumatic brain injury, randomized controlled trial, mixed methods, joint display

## Abstract

Background: The long-term consequences of traumatic brain injury can create major barriers to community integration. Peer support represents a sustainable model of support across this transition. The objective of the current study was to determine the feasibility of conducting a randomized controlled trial on the Ontario Brain Injury Association Peer Support Program and the preliminary effectiveness of the program on community integration, mood, health-related quality of life, and self-efficacy; Methods: A pilot feasibility randomized controlled trial with an embedded qualitative component was conducted. Mentees with moderate-to-severe traumatic brain injury (n = 13) were randomized to a weekly intervention or waitlist control group. Interviews were conducted with a subset of mentees and peer mentors (n = 10). Integration of the quantitative and qualitative data was completed using a joint display approach; Results: No statistically significant results were found for community integration, mood, or self-efficacy; however, changes in these outcomes were accompanied by moderate-to-large effect sizes. Within health-related quality of life, the mean pain score of the intervention group was significantly lower than that of the control group at the two-month timepoint but not at completion. Interviews revealed proximal improvements in knowledge, skills, and goals, and identified two domains related to trial acceptability: (1) environmental context and resources, and (2) reinforcement; Conclusions: Given the conceivable importance of proximal improvements in domains such as knowledge, skills, and/or goals for the attainment of more distal outcomes, modifications to the existing Peer Support Program may be warranted. The introduction of program recommendations which promote discussion around particular domains may help facilitate long-term improvements in health outcomes.

## 1. Introduction

Traumatic brain injury (TBI) causes severe disability in 150–200 people per million each year and may cause long-term physical, behavioral, cognitive, and emotional impairments [1,2]. These impairments can decrease social contacts and leisure activities, disrupt relationships, and prevent an individual from returning to employment, thus creating major barriers to post-injury community integration [2]. In addition to the individual consequences of TBI, there may also be an impact on the family members of individuals with TBI [3]. Caregivers and/or family members are frequently relied upon for general assistance, socialization, and long-term support [3]. Changes in behavior (e.g., increased aggression and irritability), cognition (e.g., memory problems), and emotional self-regulation in individuals with TBI have been described as the most common sources of burden for families [3].

Rehabilitation programs aim to restore abilities, reduce social isolation, and provide the necessary adaptive skills for individuals with TBI to resume their activities of daily living [4]. Improving the integration of adults with TBI into the community has been identified as a primary goal of rehabilitation, with social integration and quality of life suggested as areas for particular focus [5]. Screening for, assessing, and treating other complications such as anxiety following moderate-to-severe TBI are also important, as clinically significant anxiety (i.e., anxiety which can interfere with activities of daily living) has been reported in individuals with TBI 12 months after injury [6].

While healthcare professionals are experts in the management of health conditions and the functional rehabilitation of people living with brain injury, individuals with brain injury have first-hand understanding of and insight into the experience of living life with brain injury. Peer support is defined as the provision of support by an individual with experiential knowledge (i.e., peer mentor) who has similar characteristics to the recipient or mentee [7]. Mentees often consider their peer mentors to be positive role models [7,8,9]. Peer support includes the delivery of some degree of each of three types of support: emotional (i.e., caring and encouragement to counter self-esteem threats), informational (i.e., delivery of relevant information for problem-solving), and appraisal (i.e., affirmation and reassurance) [7]. This method of support has emerged as a promising alternate intervention for concurrent use with other treatments, and as an independent solution to many of the health system gaps present in the current healthcare context (e.g., difficulty in accessing specialist care) [10,11].

Previous studies have demonstrated the clinical effectiveness and cost-effectiveness of peer support interventions across a variety of chronic conditions and circumstances [7,8,9,12,13,14,15,16,17,18,19,20,21,22]. However, many of these studies discuss the need for further research on the adaptation of interventions to the needs of individuals, populations, and settings, as well as the optimal implementation of specific intervention components and sustainability of intervention outcomes. Recent systematic reviews which have been conducted on peer support for individuals with brain injury have focused on acquired brain injury, one-to-one peer mentoring, and peer support groups, with further investigation of specific intervention characteristics such as dosage, length, and communication type recommended [4,23,24,25]. A systematic review conducted by members of our research team showed a notable absence of underlying theories supporting the majority of interventions [24].

Despite a lack of intervention trials examining peer support interventions for individuals with TBI within Canada, the Ontario Brain Injury Association (OBIA) has offered a peer support program since 2006 [26]. The OBIA Peer Support Program is modelled after the mentoring partnership program used by the New York and New Jersey Brain Injury Associations which was derived from the Parent to Parent program [26]. The program is the sole formal peer support program available for individuals with TBI in the province and is made available to individuals with TBI at no cost.

Peer mentors are individuals with a brain injury who offer guidance and support to mentees based on their personal experience. Successful peer mentor candidates are identified using criteria provided by the OBIA, including the evaluation of the character traits of a good mentor, consideration of conceivable red flags, and character reference checks. Peer mentors subsequently complete a one-day group training session covering a variety of relevant topics (e.g., applying effective mentoring skills, recognizing enablers and barriers of communication) [26].

The OBIA Peer Support Program matches mentees with peer mentors for a series of one-on-one interactions. Matches may be made based on factors such as life circumstances, needs, marital status, mechanism of injury, personal interests and/or gender [27]. Communication between mentees and peer mentors primarily occurs via weekly telephone calls which typically last between 20 and 40 min. Conversations focus on the discussion and resolution of issues related to topics including the brain injury itself, challenges or life changes, emotions and feelings, employment and volunteering, family and friends, healthcare professionals, and/or social and recreational activities. Communication continues until problems are resolved, or until a referral to another service (e.g., counselling, community support services) is made. A 2012 pre–post evaluation of the OBIA Peer Support Program by the Ontario Neurotrauma Foundation which focused on mentees and peer mentors with all types of acquired brain injury called for the further study of the matching process between mentees and peer mentors, the outcome measures selected for the program, and the indirect impact of the program on relatives, as well as for a more rigorous evaluation of the program via a randomized controlled trial (RCT) [28].

Peer support programs may be rooted in theoretical frameworks such as Bandura’s social cognitive theory and its theoretical concept of self-efficacy [12]. Self-efficacy is an early step in the causal pathway of behavior change and increasing it is necessary for behavior change [12,29]. Thus, mechanisms such as using the mastery experience of others, role modeling, social persuasion, and reframing of physiological and affective states, which all affect self-efficacy, may serve to support the behavior changes of participants in peer support programs by using peer mentors as a gateway for increasing self-efficacy [12]. For the OBIA Peer Support Program, the exact active ingredients are unknown as the OBIA does not purport specific theoretical underpinnings for its intervention. The active ingredient(s) of the OBIA Peer Support Program which lead to behavior change would be important to determine for a future, large scale evaluation.

The overall objective of this study was to determine the feasibility of conducting an RCT and the preliminary effectiveness of the OBIA Peer Support Program. The specific objectives of this research are to (1) evaluate the feasibility of mentee participant recruitment and retention, data collection, and mentee participant adherence to the OBIA Peer Support Program, as well as the effectiveness of the program compared with a waitlist control group on community integration (primary outcome), mood, quality of life, and self-efficacy; (2) understand the impact and acceptability of the program from the perspective of mentees and peer mentors. Results from the current study are expected to inform the development of a large scale RCT on the impact of peer support for individuals with moderate-to-severe TBI as well as future iterations of the OBIA Peer Support Program, including potential revisions to the curriculum.

## 2. Materials and Methods

This study was registered at ClinicalTrials.gov (ClinicalTrials.gov Identifier: NCT03450460) and was conducted between May 2018 and July 2020. Written informed consent was obtained from all mentees and peer mentors prior to participation. Ethics approval was granted by the University Health Network Research Ethics Board (protocol numbers 18-5382 and 19-5107).

### 2.1. Study Design

We conducted a pilot feasibility RCT with an embedded qualitative component as described by Alicia O’Cathain [30]. This design can aid in the explanation of RCT results and generation of hypotheses regarding the mechanisms of action of an intervention [30]. An integrated knowledge translation (IKT) approach guided the overall study and helped finalize the protocol for the RCT [29,31]. The pilot feasibility RCT was reported in accordance with the Consolidated Standards of Reporting Trials (CONSORT) guidelines, while the qualitative component followed a qualitative descriptive approach and data were reported in accordance with the Consolidated Criteria for Reporting Qualitative Research (COREQ) guidelines [32,33].

### 2.2. Participants

Eligible mentee participants met the following inclusion criteria: (1) were community-based (i.e., no longer participating in a comprehensive rehabilitation program); (2) had moderate-to-severe TBI (defined as a score of less than 12 on the Glasgow Coma Scale [34], or loss of consciousness and admittance to a hospital); (3) were 18 years of age or older; (4) were fluent in English; (5) were able to provide informed consent or had an available proxy to provide informed consent. Mentee participants were excluded if they (1) had previously participated in the OBIA Peer Support Program or were currently part of another peer support or self-management program; (2) were medically unstable; (3) had active suicidal ideation. Peer mentor participants adhered to the same inclusion and exclusion criteria as mentee participants but were permitted to have had previous mentorship experience with the OBIA Peer Support Program.

Mentee participants were recruited by peer support program coordinators through the existing intake process for the program. In addition, an online advertisement was posted on the OBIA website as well as the websites of 15 participating local brain injury associations. Targeted recruitment strategies, including outreach to clinic physicians and teams to increase awareness of the study, University Health Network researchers to advertise the study to their past study participants, and key acquired brain injury contacts within Ontario who could advertise the recruitment flyer, were adopted. Recruitment for the RCT took place between December 2018 and May 2020. As mentees and peer mentors in the intervention arm of the RCT completed the study, they were recruited for the follow-up qualitative component (between August 2019 and June 2020).

### 2.3. Intervention

Once a mentee was enrolled in the study and assigned a study group, the peer support coordinator would match the mentees in the intervention group with a peer mentor. These peer mentors were then invited to participate in the study. The study lasted four months and mentees were randomized to one of two groups: a once per week intervention or a waitlist control group. In the intervention group, mentees received the OBIA Peer Support Program as soon as they were able to be matched with a mentor. The focus of the intervention was unidirectional and on the mentee. Mentee-mentor pairs had the flexibility to change the length and/or frequency of their meetings based on individual needs and/or preferences. Individuals assigned to the waitlist control group received the OBIA Peer Support Program after completing their time in the study. As the OBIA Peer Support Program already maintains a waitlist, this assignment did not vary substantially from the typical intake procedure and was deemed acceptable by the OBIA [31].

### 2.4. Randomization and Blinding

The random allocation sequence was generated using a web-based randomization service by a research coordinator who was not involved in quantitative data collection, outcome assessment, or data analysis. Mentee participants were randomized to either the intervention or waitlist control group in a 1:1 ratio with random block sizes of 2 and 4. The allocation sequence was concealed using sequentially numbered, sealed, opaque envelopes until mentee participants were assigned to a group. The research coordinator enrolled and assigned mentee participants to interventions. The nature of the intervention did not permit the blinding of mentees and mentors; however, outcome assessment and data analysis were both blinded.

### 2.5. Outcome Measures for the Pilot Feasibility Randomized Controlled Trial

#### 2.5.1. Feasibility Outcomes

Study recruitment, adherence, and retention were measured. The feasibility of recruitment was based on the proportion of individuals who were enrolled compared to those individuals who were approached (and were eligible). The proportion of weekly sessions attended by mentees was calculated to assess adherence. The feasibility of retention was evaluated by the proportion of mentees with complete data on each outcome measure at two and four months. The proportion of mentees who withdrew from the intervention at two and four months was also calculated, with the reason(s) for withdrawing noted.

#### 2.5.2. Community Integration (Primary Outcome)

Community integration was measured using the Community Integration Questionnaire (CIQ) [35]. The CIQ consists of 15 items which assess home integration, social integration, and productive activity [35]. The instrument can be completed via self-report or with caregiver assistance [35]. The overall CIQ score ranges from 0 to 29, with a higher score being indicative of a higher level of community integration [35]. For individuals with TBI, the test–retest reliability coefficient for all subscales of the CIQ is 0.91 [35].

#### 2.5.3. Mood (Secondary Outcome)

Mood (i.e., depressive symptoms) was measured using the Patient Health Questionnaire (PHQ)-9 (i.e., the nine-item depression module from the PHQ) [36]. PHQ-9 scores range from 0 to 27, with each of the nine items self-reported on a scale from 0 to 3 [36]. A higher score is indicative of a higher level of depressive symptoms over the previous two weeks [36]. Scores are divided into minimal (0–4); mild (5–9); moderate (10–14); moderately severe (15–19); severe (20–27) depression severity. Clinical significance of self-reported symptoms of depression is denoted by a score equal to or greater than 10 [37].

#### 2.5.4. Health-Related Quality of Life (Secondary Outcome)

Health-related quality of life was measured using the self-administered Medical Outcomes Study Short Form-20 (SF-20) [38]. The SF-20 consists of 20 items and assesses six health concepts: physical functioning, role functioning, social functioning, mental health, health perceptions, and pain [38]. Scores are reported from 0 to 100 for each of the six health concepts, with a higher score indicative of better health (except for pain, for which a higher score is indicative of more pain) [38].

#### 2.5.5. Self-Efficacy (Secondary Outcome)

Self-efficacy was measured using the TBI Self-efficacy Questionnaire (TBI-SE) [39]. This instrument contains four subscales: social, cognitive, emotional, and physical [39]. These subscales measure self-efficacy for obtaining help for activities of daily living as well as emotional support; managing and compensating for cognitive symptoms; managing emotional symptoms; managing and compensating for physical symptoms, respectively [39]. Items are self-rated on a scale from 1 to 10 where 1 represents “not at all confident” and 10 represents “totally confident” and are summed to generate a total score from 26 to 130 [39].

### 2.6. Data Collection and Analysis

#### 2.6.1. Pilot Feasibility Randomized Controlled Trial (Quantitative Component)

Quantitative data collection was completed at baseline, two months, and four months for mentees randomized to the intervention group. Data were collected via telephone and mentees were given the option to have a caregiver or family member present during the administration of survey questionnaires.

As the current study was a pilot feasibility RCT study [40] and tested the ability to collect data, no data imputation was performed to account for missing data. A formal sample size calculation was also not performed as one of the specific objectives of the current study was to evaluate the feasibility of participant recruitment and retention [29]. A target of 20 individuals per trial arm was initially set based on a review of previous pilot and feasibility trials; however, participation in the current trial was lower than anticipated [29,41].

Demographic and clinical characteristics and outcome information at each time-point for each partner group were reported as mean (standard deviation) or median values (interquartile range) for continuous variables and as counts (percentages) for categorical variables. Further, a descriptive summary of mentor demographics was also presented via counts and percentages. Differences between partner groups on demographic and clinical characteristics were examined using independent samples *t*-tests for continuous variables and chi-square tests for categorical variables. A series of repeated measures analysis of variance models (RM ANOVA) were applied to examine differences in outcomes at baseline and four months post-intervention between partner groups. Normality assumptions were visually assessed via histogram plots, skewness and kurtosis values, and tested using Shapiro Wilk’s test. Homogeneity of variances and covariances were assessed using Levene’s test and Box’s M test, respectively. Where applicable, violations of these assumptions were reported for transparency. Hedge’s *g* effect size, which is recommended for small sample sizes, was also reported to examine group mean differences at four months post-intervention [42]. Hedge’s *g* is a non-parametric effect size that can be calculated for groups with different sample sizes [43]. Given the exploratory nature of this work, these effect size calculations allow for examination of the magnitude of mean differences between groups, and also facilitate a priori power analyses for future research studies in this area [42]. To compute Hedge’s *g*, means, standard deviations, and group sizes were entered into an effect size calculator to provide a Hedge’s *g* values and corresponding 95% confidence intervals. Hedge’s *g* values of 0.2, 0.5 and 0.8 reflect small, moderate and large effect sizes, respectively. All statistical tests were two-sided and an alpha of 0.05 considered statistically significant. Analyses were conducted using SPSS version 25.

#### 2.6.2. Interviews (Qualitative Component)

Mentees randomized to the intervention group and their mentors completed one-on-one, semistructured telephone interviews which lasted approximately 45–60 min. The research coordinator for the project (DL) conducted all of the interviews. DL is a female and has a MSc in Rehabilitation Science with 12 years of experience in conducting qualitative studies. Mentees and mentors were asked about their experiences in the OBIA Peer Support Program, including the strengths and weaknesses of the program as well as enablers and barriers to participating in the program. Suggested modifications to the current mentor training agenda were also explored with peer mentor participants. Probes and recursive questioning were used to explore issues in greater depth and confirm understanding of collected information [44].

Interviews were all digitally recorded and transcribed verbatim. Transcripts were entered into NVivo to aid in the organization and analysis of the data. The Theoretical Domains Framework (TDF)(v2) was used as the primary coding framework to analyze interviews (i.e., deductive approach) [45], although an inductive approach [46] was also adopted to allow for the possibility of identifying themes that were not consistent with the TDF(v2). A subset of interviews was initially coded by the principal investigator (SM) and research coordinator independently. The codes for this subset were subsequently compared to ensure enhanced reflexivity and rigor. Discussion between the coders and reference to the original transcripts were used to resolve disagreements or discrepancies around codes.

### 2.7. Integration of Data Sets

Following analysis of the both the quantitative and qualitative data, the data sets were compared to evaluate similarities and differences in the findings [47]. The data were integrated using a joint display, which is defined as a method of “[integrating] the data by bringing the data together through a visual means to draw out new insights beyond the information gained from the separate quantitative and qualitative results” [48]. The joint display aided with data interpretation as part of a more rigorous and robust analysis [49].

## 3. Results

### 3.1. Quantitative Data

Mentee demographic data and clinical characteristics at baseline are presented in Table 1. The mean age of the intervention group at baseline was determined to be significantly higher than that of the waitlist control group (*p* = 0.047) using an independent samples *t*-test. No other statistically significant differences between the intervention and control groups were determined. Peer mentor demographic data are presented in Table 2.

#### 3.1.1. Recruitment, Adherence, and Retention

**Figure 1:**
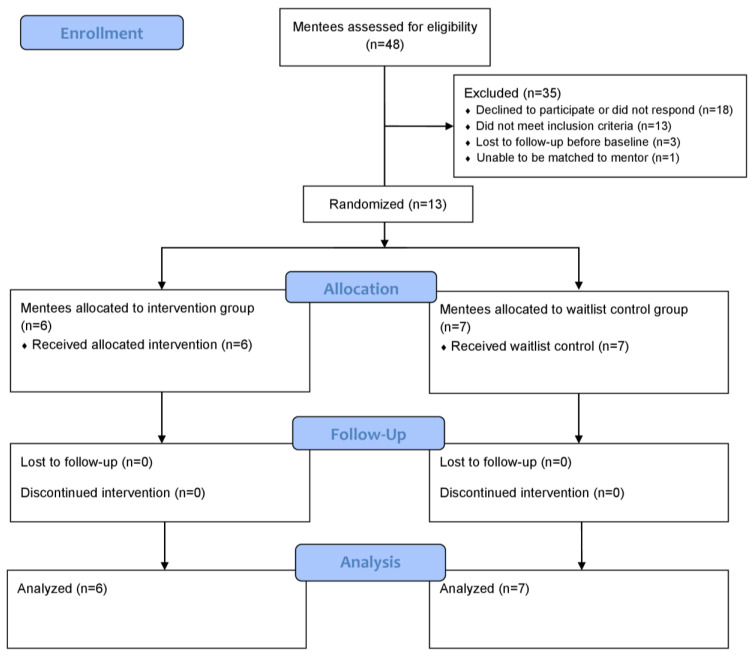
Mentee participant flow diagram.

Mentee participant flow is presented in Figure 1. In total, 13 individuals were enrolled in the RCT: six in the intervention group and seven in the control group. Intervention adherence varied from 33% of weekly sessions logged to 89% of weekly sessions logged based on weekly expected delivery of the intervention; however, two mentee–peer mentor pairs chose to adhere to a twice-per-month (i.e., biweekly) schedule. The average call length was 51 min. Total sessions and average session length for mentee–peer mentor pairs are presented in Table 3. All six mentee–peer mentor pairs completed the intervention, but baseline data for one pair were missing as the researchers were only informed of this match after weekly correspondence had started. Of the six mentees and six peer mentor participants in the intervention group, five mentees and five peer mentors participated in the qualitative component.

#### 3.1.2. Community Integration

Community integration mixed ANOVA data are presented in Table 4. Raw outcome measure data are available as a Supplementary Materials File (Table S1). There was a non-statistically significant decrease in the intervention group’s mean overall CIQ score between baseline and intervention completion with a moderate effect size (*g* = 0.617; CI: −0.639–1.872). No significant main effects for time or group were observed for any of the subscales or for overall score. No significant interactions were found for any subscale or overall score.

#### 3.1.3. Mood

Mixed ANOVA data for mood are presented in Table 5. Raw outcome measure data are available as a Supplementary Materials file (Table S2). The intervention group experienced an initial non-statistically significant worsening of mood at the two-month time point followed by a non-statistically significant improvement relative to baseline at the four-month time point with a moderate effect size (*g* = 0.447; CI: −0.715–1.608). The data on mood met the criteria for normality and homogeneity of covariances and variances; however, no significant main effects for time or group were observed. No significant interaction was observed.

#### 3.1.4. Health-Related Quality of Life

Health-related quality of life mixed ANOVA data are presented in Table 6. Raw outcome measure data are available as a Supplementary Materials file (Table S3). The intervention group had a significantly lower mean pain health concept score than the control group at two months (*p* = 0.021; *g* = 0.187; CI: −0.905–1.28); however, this result was not maintained at four months and pain data violated the assumption of homogeneity of variances. The mean role functioning health concept score significantly increased from baseline to four months for both the intervention and control groups (*p* = 0.05; *g* = −0.242; CI: −1.337–0.852); however, no interaction was observed and the data on role functioning violated the assumption of normality. No other assumption violations were observed.

#### 3.1.5. Self-Efficacy

Self-efficacy mixed ANOVA data are presented in Table 7. Raw outcome measure data are available as a Supplementary Materials file (Table S4). There was a non-statistically significant decrease in the intervention group’s mean total self-efficacy score between baseline and intervention completion with a moderate effect size (*g* = 0.585; CI: −0.529–1.698). No significant main effects for time or group were observed for any self-efficacy subscale. A trend toward significance for an interaction with a large effect size was found for the social and community subscale, with the intervention group showing a greater decline in this subscale than the control group at the four-month time point (*p* = 0.063; g = 1.005; CI: −0.152–2.161). Assumptions of homogeneity of variances, covariances, and normality were met for all models.

### 3.2. Qualitative Data

#### 3.2.1. Outcomes of Participating in Peer Support

Interview themes encompassed nine domains from the TDF(v2) which were related to the outcomes of participating in the OBIA Peer Support Program. These domains included knowledge, skills, beliefs about capabilities, optimism, beliefs about consequences, reinforcement, goals, social influences, and emotion.

##### Knowledge

Both mentees and peer mentors described acquiring knowledge as an outcome, with many of the components relating to improved knowledge of self-management. This included receiving and sharing knowledge of resources as well as tips around strategies for symptom management, medications, pain management, legal processes, and other issues related to brain injury. When asked about the most helpful topics of conversation, one mentee stated:

*I think talking about medications, like strategies and tools that we use to deal with our anxiety, our depression, our post-trauma, our stress. And, the fact that it’s a chronic thing. And our pain. Physical pain. My knee, his knee as well. Strategies, concrete things, a crutch or a wheelchair or prosthetic knee, that he uses.*

(ID #1006)

In addition to newly acquired knowledge, this domain also encompassed reminders of existing knowledge (e.g., a reminder about a certain available resource with subsequent usage of that resource). One mentee said about her peer mentor:

*I wouldn’t necessarily say that she gave me more of an education but she reminded me about things that maybe I had forgotten about or that I stopped doing. Like vitamins for example, like I used to take omega-3 and I stopped.... so she kind of reminded me about that and now I’ve started taking the vitamins again which I found helpful before and now I’m finding helpful again.*

(ID #1005)

##### Skills

Both mentees and peer mentors described the development of skills as an achieved outcome. Specifically, knowledge sharing on certain issues promoted skill development (e.g., returning to work, dealing with exhaustion and memory issues, and organizational strategies such as using calendars and calendar reminders). With respect to concrete organizational strategies, one mentee stated:

*… [my peer mentor] also gave me some suggestions with organization in my house because we do a lot of laundry with little kids and she suggested these laundry tote containers.... she suggested these totes that were really helpful for her to organize her kids’ stuff. I was able to get hold of some and that helped me organize the laundry better and that was helpful.*

(ID #1005)

Mentees and peer mentors also both described interpersonal skills development, including improved phone skills, as an outcome of participating in the program. Peer mentors discussed improvements in several mentor-related skills, including improved listening skills, selflessness, and increased ability to have difficult conversations: “*The Peer Support Program has changed my life to be a better person, be more understanding, be more open, and it also teaches the value of what one smile can do, communication and just to have a conversation that I used to take for granted.*” (ID #1001)

##### Beliefs about Capabilities

Both mentees and peer mentors indicated that they experienced self-confidence, perceived competence, self-efficacy, and empowerment as outcomes. Mentees shared that they felt more confident in decisions or actions that they had taken due to validation from peer mentors:

*I know on several occasions there were things that I spoke to my mentor about, that were really helpful that I wasn’t feeling confident about… just stuff with the kids, like stuff where I thought maybe I was not doing a great job and I talked to [my peer mentor] and she was very validating… I know that that happened on several occasions that it was after talking with her it was just really helpful and reassuring.*

(ID #1005)

Peer mentors reported increased confidence as a result of providing help to others via their participation in the program:

*If you don’t have confidence in yourself, it’s going to make it more difficult. So, for me the confidence is a big issue and the program helped me do that.... I think confidence is a big issue. So, when you’re confident in talking to others, then it might help you a lot. The program helped me do that.*

(ID #1001)

This peer mentor further spoke about pushing himself to become more confident in the way he lived his life with the understanding that mentees would want a peer mentor who is confident during discussions. Mentees also spoke about how increased confidence related to better overall mood.

##### Optimism

Optimism was identified as an outcome or intended outcome for mentees, particularly if they were at an earlier stage in their recovery. Mentees described a sense of hope in terms of progress in their recovery by looking to their peer mentors. As one peer mentor described:

*When I was a mentee, the one-on-one that I had with my mentor was so critical, because the issues were very specific to me and I needed somebody that [was] further along in the journey to help me figure it out. Like, “what’s going on.” Also, looking to them as hope, that I can get to wherever they are in that healing stage.*

(ID #1009)

##### Beliefs about Consequences

Mentees described experiencing a shift in their beliefs about consequences and peer mentors stated that reframing beliefs about consequences was a frequent topic of conversation. For example, peer mentors helped mentees realize that the recovery process is not necessarily about going back to how things were before the injury:

*What I try to do is not to let them look back and talk about what they used to do before. My goal is try to go forward, things that they can do now.... People always are in awe about what they used to do before and they’re trying to get back their life in full. And it doesn’t work like that, because I used to do the same thing.... The brain cannot be rushed. It takes its own time.*

(ID #1001)

One mentee described being open to not fitting the standard narrative of “recovery” as defined by therapists:

*There’s this narrative that’s like, get the person back to work and at all costs, no matter what their quality of life or whatever. It’s all about work. And I appreciate that work is so much of our sense of identity before we’re injured. And it took me until I settled, so probably seven or eight years, to accept the fact that I wasn’t going to get an identity through working. And to give up on that narrative that all the therapists were pushing. I’m a lot happier now that I’m not following this narrative of what the therapists say.*

(ID #1008)

##### Reinforcement

Reinforcement was experienced as an outcome by mentees and peer mentors and most frequently described as validation. Reinforcement was closely related to social support and the sense of validation gained from speaking to someone with similar experiences as one’s own. Mentees’ perceived benefits also reinforced their participation:

*It’s increased [my behavior and thinking and knowledge]. My mood, like I said. My motivation, my energy. Because, it’s a pleasant phone call. It improved my social [sic.]. I’m less anxious to talk to people on the phone. I’m more comfortable. I like it. I enjoy it. I enjoy the process.*

(ID #1006)

##### Goals

Both mentees and peer mentors described goals and goal setting as components of the program. For example, for one partner, becoming a peer mentor was a goal upon completion of the program: “*I thought that [becoming a peer mentor] might be a good goal to set for myself, probably not right now but at some point in the future…*” (ID #1005). Peer mentors described the realization of their own shortcomings (e.g., not being more involved in the community or volunteering) when speaking with mentees, as well as the need to set their own goals: “*[Being a peer mentor] also helps me realize some of my challenges and some of the things I need to work on.... Things I need to work on to set goals for.*” (ID #1007). Peer mentors also discussed goal setting as a topic of conversation with mentees:

*“My goal is to try to go forward, things that they can do now.... because the main thing with brain injury I find that people always look back.... So, I try to let them look forward, not to forget about their life, but they’ve got to put it on the back burner and focus on this now”.*

(ID #1001)

Personal short-term goals, such as what a mentee was “*hoping to do the following week*”, (ID #1007) were noted as specific discussion points.

##### Social Influences

Social support arose as a dominant theme in the interviews and was experienced by both mentees and peer mentors as an outcome. Mentees and peer mentors described feeling validated from speaking to someone who had been through a similar experience and from the mutual sharing of similar experiences, which provided social connection and a group identity:

*It was nice to know that there was [sic.] other people and that you’re not in your own little bubble by yourself and your issues are legitimate. It’s nice to have that social piece with people that really understand and are going through similar situations or at least have been through similar situations.*

(ID #1005)

The construct of social comparisons was also cited as a reason for mentee participation, and hearing about the successes of a peer mentor with a similar experience served as a form of validation for mentees.

The stage of the relationship and time since injury were also considerations that arose in this theme. These considerations impacted the length and frequency of calls (e.g., longer and more frequent calls earlier in the relationship), as well as the nature of the conversations and the type of support peer mentors felt they were able to provide (e.g., conversations around knowledge and strategies for symptom management were often had with mentees earlier in their recovery). The importance of finding the “right” mentee–peer mentor match was also considered an important facilitator to implementation of peer support and relationship development. Important matching characteristics included severity of injury and similarities in profession, relationships, and personalities.

##### Emotion

Both positive and negative emotions were experienced by mentees and peer mentors. Positive mood was described as feeling better after talking to someone. Negative mood, stress, and negative affect were also described as outcomes for both mentees and peer mentors, particularly following negative or depressing conversations. Negative affect was experienced by peer mentors as a result of difficult conversations or substantial emotional investment in a mentee; feelings of unfulfillment due to an unsuitable match; frustration with organization-level issues which had not been addressed, such as long wait times for a match. When speaking about match suitability and its impact on emotion, one peer mentor stated:

*When I was partnered as the mentee, I really enjoyed that experience and I really liked both of my mentors. I felt it was easy to talk to the person, we had lots of things in common. Our conversations, generally speaking, were never boring. We would always talk for an hour, at least. Or it felt like that, unless one of us was sick. So, I just assumed that’s what it was going to be like when I got my own mentee, but it was the opposite of that. The first couple of conversations were good and since then it’s just been, I don’t know, we don’t have much in common. I guess I’m just a bit disappointed.*

(ID #1009)

#### 3.2.2. Acceptability of the Randomized Controlled Trial

Interview questions also focused on acceptability of the trial itself. Two domains emerged in this area: (1) reinforcement; (2) environmental context and resources.

##### Reinforcement

Incentives for participation related to a sense of giving back via participating in research or the possibility of benefitting in some way from the research. One mentor stated, “*The other bonuses [sic.], I have already mentioned, that I can be part of making things and improving things for people with brain injury.*” (ID #1003). Monetary incentives were not the primary factor for participating in research and were described as a bonus. Peer mentor participation and satisfaction were facilitated by a variety of factors, including the need for peer mentors to get feedback on their performance; the drive to “*serve somebody else and just be there for them*” (ID #1009) even if the mentorship was not progressing in the expected or intended manner; gaining benefits such as enhanced social connection from the program.

##### Environmental Context and Resources

Mentees stated that researchers had provided an acceptable environment (i.e., the right conditions) for conducting the study. There was some preference for face-to-face data collection by interviewees; however, data collection by phone still allowed mentees to “*… talk about [their answers] a little bit and then that way [they could] make sure that [their chosen] answer [was] the right answer.*” (ID #1010). The length of the surveys and logistics of scheduling were also deemed acceptable by interviewees. Mentees and peer mentors appreciated the flexibility in scheduling their telephone surveys, and indicated that factors such as energy and focus on a given day affected or could have affected their ability to participate in research in a meaningful way:

*I think that because of my lack of energy maybe the quality of some of my answers towards the end weren’t as accurate or effective just [sic.]. Because I was so tired maybe I wasn’t giving the most accurate answers, if that makes sense.... And obviously I know it’s hard to break things up but for people that are recently injured that’s a really tricky piece, is the energy piece, the energy component.*

(ID #1005)

Mentees indicated that their responses to survey items might be highly variable even during a single day, with one respondent wondering if the research was accurately capturing outcomes.

#### 3.2.3. Integration of Quantitative and Qualitative Results

Our joint display (Table 8) presents the quantitative and qualitative data for each main outcome of the current study, evaluates the degree of complementarity and/or contradiction between these data, and provides an integrated analysis of these data. Our integration of the quantitative and qualitative data revealed patterns of (1) some complementarity; (2) some complementarity and some contradiction; (3) contradiction, distributed across each of the main study outcomes (i.e., feasibility, community integration, mood, health-related quality of life, and self-efficacy).

## 4. Discussion

### 4.1. Feasibility of Conducting an RCT of the OBIA Peer Support Program

A specific objective of the current study was to evaluate the feasibility of mentee participant recruitment and retention, data collection, and mentee participant adherence to the OBIA Peer Support Program. While the research team did face several recruitment challenges (i.e., only 13 out of the initial 48 individuals assessed for eligibility were ultimately allocated to the intervention or control group and participated in the trial), data collection (except for in one outlier circumstance at baseline) was fully completed by all mentee–peer mentor pairs. The majority of excluded individuals either declined to participate or did not respond to researchers or did not meet the inclusion criteria for the current study given the focus on moderate-to-severe TBI. Scaling recruitment efforts for a future RCT may be necessary given the difficulties experienced with recruiting individuals with moderate-to-severe TBI, who comprise only a subset of the entire TBI population. The addition of a dedicated individual to assist with province-wide recruitment and research efforts, as well as the adoption of more consistent contacts as part of the recruitment process, are also conceivable strategies to offset recruitment difficulties.

The personalized nature of the program and the one-on-one relationships fostered between mentees and their peer mentors throughout the program’s duration are conceivably factors which promote mentee participant retention; retention was at 100% in the current study. As the relationships between mentees and peer mentors naturally strengthen over time, both mentees and peer mentors may also begin to feel more committed to the program and accountable for being available for scheduled calls. If mentees or peer mentors are earlier in their recovery and/or have expectations of gaining specific benefits (e.g., discussing specific topics) from participating in the program, introducing specific recommendations with themes and/or topics for discussion each week (alongside open discussion) may also help to promote adherence. Based on mentee and peer mentor feedback, the flexibility associated with outcome measurement scheduling was an important factor which contributed to complete data collection in the current RCT. Therefore, data collection processes in future RCTs should remain flexible for mentees and peer mentors and highlights the value of our IKT approach in terms of refining the trial protocol [31]. Future RCTs should also continue incentivizing participation by (1) promoting the notion that participating in research is a way of contributing to one’s community; (2) allowing peer mentors to gain feedback and social connections from the program, and/or; (3) using monetary benefits.

### 4.2. Similarity of Results from the OBIA Peer Support Program to Previous Studies

Previous systematic reviews of peer support interventions for brain injury have examined peer support for the rehabilitation of individuals with acquired brain injury [23]; one-to-one peer mentoring for people with TBI [4], and; peer support groups following acquired brain injury [25]. However, the effects of peer support for individuals with TBI have only been studied in two other RCTs [24]: a 2012 study by Hanks et al. [50] which included peer mentoring for individuals with TBI (n = 96) and their caregivers or significant others (n = 62), and a 2011 study by Struchen et al. [51] which examined a social peer mentoring program for individuals with TBI (n = 30).

In the study by Hanks et al., the TBI intervention group showed no significant improvement in community integration compared to the TBI control group following a mentorship period of up to two years [50]. Moreover, the significant others who were mentored in the program showed significantly less community integration than the non-mentored control group [50]. However, the TBI intervention group did demonstrate significantly less emotion-focused (i.e., blame-focused) coping and better physical health-related quality of life compared to the TBI control group [50]. This finding underscores the hypothesis that improvements in community integration for individuals with TBI may occur distally and need to be preceded by improvements in other, more proximal outcomes. Similar to the current study, the majority of participants did report a positive experience with the peer mentoring program in the study by Hanks et al. [50].

In the study by Struchen et al., participants were also highly satisfied with the social peer-mentoring program and additionally showed a trend toward increased satisfaction with social life following the three-month intervention [51]. However, there were no significant improvements found in social activity level or social network size for mentored participants and a significant increase in depressive symptoms was also found in this group [51]. This increase in depressive symptoms mirrors the initial worsening of mood observed at the two-month time point in the current study. While subsequent improvement in depressive symptoms (with moderate effect size) was observed at final outcome assessment in the current study, successful management of the cyclical nature of mood throughout the duration of a peer support intervention may require more advanced peer mentor training around handling difficult conversations (e.g., recognizing and shifting mood during conversations). Our data integration also suggests that the inclusion of recommendations around knowledge, skills, and/or goals during conversations may help with the balancing of positive and negative emotions for both mentees and peer mentors.

### 4.3. Recommendations for Future Implementation of the OBIA Peer Support Program

Our data identify the conceivable importance of proximal improvements in domains such as knowledge, skills, and/or goals for the attainment of more distal outcomes (e.g., community integration). Therefore, several modifications to the existing OBIA Peer Support Program may be warranted, including the introduction of a set of recommendations for mentees and peer mentors which encourage discussion around particular domains before completion. This may help facilitate early improvements in domains which are believed to serve as vehicles for the ultimate improvement of outcomes including self-efficacy, community integration, and health-related quality of life. Such recommendations might involve the inclusion of brief, weekly check-ins around (1) the resources which a mentee has reviewed and/or used to improve knowledge; (2) progress in a mentee’s skill development, and/or; (3) assisting in setting or attaining goals, especially as the partnership grows and/or if the mentee expresses a desire for setting or attaining goals. At the same time, some mentees may feel relief that they can “just talk” in this partnership without the pressure of goal setting. Thus, the successful implementation of goal setting as part of the partnership would be dependent on the comfort of both the mentee and peer mentor.

Several peer mentors elaborated on their own experiences of community integration in the interviews, which further supports the notion of community integration being a long-term outcome which mentees will develop over time, similar to their peer mentors. Vicarious experience, described in Bandura’s self-efficacy theory as seeing or hearing about an experience of another and feeling this experience, arose as a component of several themes identified in the interviews, including beliefs about capabilities, social influences, and reinforcement, which was described as validation [12]. These were important elements of the peer support program, as they served to guide mentees’ expectations for future success and may have been a contributing factor to mentees’ early development of knowledge and skills [12]. In addition to using vicarious experience, self-efficacy may also be enhanced via continued reinforcement from peer mentors or with a focus on skill mastery [12]. However, irrespective of the specific framework used, intervention development should be guided by a systematic approach as interventions informed by theory may lead to better outcomes [52].

Recommendations (e.g., a playbook for mentees and peer mentors) may also help to better equip mentees to handle difficulties which may arise in the future, following the completion of a peer support program. While a mentee may not be immediately struggling in a given area (e.g., pain) during the program, some discussion of the areas pertaining to each of the outcomes of the current study (i.e., community integration subscales, mood, the health concepts comprising health-related quality of life, and self-efficacy subscales) could be encouraged in the future. Mentees may appreciate that although they are not currently experiencing a given issue, having some brief discussions on subject areas beyond their immediate needs may allow them to better recognize other issues if they do arise. Such conversations could comprise a small component of regular sessions in order to preserve an empathetic, flexible, and person-centered approach to peer support, while also allowing mentees to work on gaining the requisite knowledge and skills to appropriately address potential challenges in the future.

### 4.4. Challenges Associated with Program Modifications and Future Outcome Measurement in Peer Support Studies

It is also important to consider that introducing specific program recommendations poses new challenges for both mentees and peer mentors. The degree of flexibility granted by the current OBIA Peer Support Program with respect to the weekly topics of discussion as well as scheduling was highly appreciated by mentees. If mentees do not have immediate topics of interest which they wish to discuss with peer mentors, they may prefer to *“let [the conversation] take its course”* rather than seek out specific strategies or topics for discussion. This may contribute to peer mentor disappointment, as identified under the emotion domain of the TDF(v2). Changes to the current program structure should consequently be completed with care taken to ensure that the program still affords mentees and peer mentors the flexibility which they desire. One approach may be to introduce a timeline with a series of milestones for both mentees and peer mentors to progressively achieve as they complete the program together. A mentee–peer mentor pair may be expected to discuss a number of specific topics by, for example, at least the halfway point of the program. This approach maintains a degree of flexibility which mentees and peer mentors have become accustomed to by accommodating the needs and wants of mentees, but also embeds an opportunity for new exploration of additional subject areas within the structure of the program.

Whereas our quantitative data did not demonstrate significant improvements in overall community integration, the majority of health-related quality of life health concepts, and total self-efficacy, our qualitative data did show that certain domains, including knowledge and skills, were perceived to have improved in mentees following peer support. Measuring knowledge and skills quantitatively throughout the duration of the OBIA Peer Support Program could have helped to substantiate the hypothesis that these two domains serve as vehicles for the improvement of other, more longer-term outcomes such as community integration or self-efficacy. Within the context of the current study, quantitative improvements in knowledge and skills (if measured) would have therefore been expected at both the two- and four-month time points as precursors to improvements in other outcomes. Quantitative measurement of both knowledge and skills using validated scales and/or measurement systems (e.g., the Adaptive Behavior Assessment System-II for skills [53]) is advised for future studies which examine the impacts of peer support.

### 4.5. Strengths and Limitations

We acknowledge some strengths of the current study including the use of the CONSORT [32] and COREQ [33] guidelines for the reporting of quantitative and qualitative data, respectively. Furthermore, blinding of the investigators to group assignment, including blinding of our statistician (JV), was done wherever possible. The TDF(v2) was used as a comprehensive approach for coding the interviews, with all coding completed by two investigators (DL, SM) independently and in duplicate [45]. However, we acknowledge that our background in knowledge translation science influenced the conceptual frameworks we were exposed to as well as our knowledge and selection of the TDF(v2) for this study. Several limitations exist within the current study, including our small sample size which led to a significant difference in age between groups at baseline and resulted in an underpowered pilot RCT, hence making it more difficult to detect significant differences in outcomes between groups. Given the small sample size of the current study, our recommendations may not be generalizable to the entire provincial Peer Support Program, which includes hundreds of mentees with varying degrees of brain injury severity. Difficulties around recruitment may have also introduced the potential for selection bias, as it is conceivable that those individuals who elected to participate in the qualitative component of the study were healthier and/or had greater motivation to do so. It was also not possible to confirm with certainty whether individuals in the control group sought other, outside support (e.g., counselling services) throughout the duration of the waitlist period, especially given the cognitive and memory limitations of this group as well as the described high turnover of peer support program coordinators.

## 5. Conclusions

Our qualitative interviews identified several apparent benefits of participating in the OBIA Peer Support Program for both mentees and peer mentors, including improvements in knowledge and skills. However, the qualitative outcomes of the current study were not all supported by our quantitative data. While aspects of the feasibility component of the trial, such as adherence and retention, showed positive results, the results from the quantitative pilot component of the trial were weaker. Focusing on attaining proximal outcomes as part of an effective peer support program may serve an important role in achieving distal outcomes such as community integration or health-related quality of life, as proximal improvements conceivably serve as vehicles for long-term improvements. The use of specific recommendations with themes and/or topics for discussion each week (i.e., a topic checklist) may help to ensure proximal outcomes are attained, as well as sustain outcome improvements over longer periods of time during an intervention (and beyond its completion).

## Figures and Tables

**Table 1:** Mentee demographic data and clinical characteristics at baseline.

Demographic Characteristic	Intervention Group, *n* = 6	Waitlist Control Group, *n* = 7	*p*-Value
*Sex, n (%)*			0.853 ^a^
Male	4 (66.7)	5 (71.4)	
Female	2 (33.3)	2 (28.6)	
*Age (years)*			0.047 ^b^
Mean/SD	50.8 (12.92)	35.6 (11.70)	
Median/IQR	49.5 (24)	32 (19)	
*Marital status, n (%)*			0.422 ^a^
Married/common	3 (50.0)	3 (42.9)	
Single/never married	2 (33.3)	4 (57.1)	
Separated/divorced	1 (16.7)	0 (0.0)	
*Highest level of education, n (%)*			0.345 ^a^
Some high school or less	0 (0.0)	1 (14.3)	
High school	1 (16.7)	0 (0.0)	
Some college/CEGEP/trade school	1 (16.7)	1 (14.3)	
Graduated from college/CEGEP/trade school	2 (33.3)	0 (0.0)	
University undergraduate degree	2 (33.3)	1 (14.3)	
Master’s or professional degree	0 (0.0)	3 (42.9)	
*Severity of brain injury, n (%)*			0.505 ^a^
Moderate	2 (33.3)	1(14.3)	
Severe	4 (66.7)	5 (71.4)	
*Missing*	0 (0.0)	1 (14.3)	

^a^ Chi-square test. ^b^ Independent samples *t*-test. Group b is significantly younger than group a.

**Table 2:** Peer mentor demographic data at baseline.

	Peer Mentors (*n* = 6), *n* (%)
*Sex, n (%)*	
Male	3 (50.0)
Female	3 (50.0)
*Age (years)*	
Mean/SD	51.0 (8.17)
Median/IQR	53 (15)
*Marital status*	
Married/common	3 (50.0)
Single/never married	2 (33.3)
Separated/divorced	1 (16.7)
*Highest level of education*	
Some high school or less	0 (0.0)
High school	0 (0.0)
Some college/CEGEP/trade school	1 (16.7)
Graduate from college/CEGEP/trade school	2 (33.3)
University undergraduate degree	3 (50.0)
Master’s or professional degree	0 (0.0)
*Number of years as a peer mentor*	
<1 year	2 (33.3)
1–5 years	3 (50.0)
6–10 years	1 (16.7)

**Table 3:** Total sessions and average session length for mentee–peer mentor pairs.

Mentee–Peer Mentor Pair	Total Sessions	Average Session Length (Minutes)
1	16	33
2	15	53
3	7	52
4	8	68
5	6	43
6	11	60

**Table 4:** Mixed ANOVA results examining changes in community integration from baseline to completion between groups.

	*p*-Values		
Outcome	Time	Group	Interaction
CIQ home integration *	0.506	0.438	0.918
CIQ social integration	0.636	0.787	0.214
CIQ productivity **			
CIQ total	0.956	0.657	0.373

* Violated assumptions of normality at T3. Total score violated assumption of homogeneity of covariances, and social and total scores violated assumption of homogeneity of variances at T3. ** This model would not run because homogeneity of covariance was unable to compute.

**Table 5:** Mixed ANOVA results examining changes in mood from baseline to completion between groups.

PHQ9	*p*-Values		
	Time	Group	Interaction
	0.498	0.57	0.235

**Table 6:** Mixed ANOVA results examining changes in health-related quality of life from baseline to completion between groups.

	*p*-Values		
Outcome	Time	Group	Interaction
Physical functioning	0.968	0.368	0.266
Role functioning *	0.05	0.946	0.372
Social functioning	0.566	0.648	0.447
Mental health	0.955	0.79	0.332
Health perceptions	0.401	0.966	0.554
Pain **	0.142	0.411	0.317

* Violated assumption of normality. ** Violated assumption of homogeneity of variances. Bold indicates statistical significance (*p* ≤ 0.05).

**Table 7:** Mixed ANOVA results examining changes in self-efficacy from baseline to completion between groups.

	*p*-Values		
Self-Efficacy	Time	Group	Interaction
Social and community	0.205	0.309	0.063 *
Physical functioning	0.478	0.485	0.95
Cognitive functioning	0.616	0.305	0.456
Emotional regulation	0.592	0.907	0.64
Total	0.622	0.545	0.471

* Trend toward significance for an interaction was found for social and community subscale.

**Table 8:** Joint display of quantitative data, qualitative data, and integrated findings for main study outcomes.

Main Outcomes	Quantitative Data	Qualitative Data	Integrated Findings
Feasibility	*Recruitment* Of the 48 interested mentees who made contact with researchers, 13 were deemed ineligible, 18 declined or did not respond after multiple consecutive attempts at communication, and 17 consented to participate. Of these 17 mentees, three were lost to follow-up before baseline and one was unable to be matched to a peer mentor and did not start the intervention. In total, 13 mentees participated in the trial: six in the intervention and seven in the control group. *Adherence* Adherence to the intervention ranged from 6 out of 18 (33%) weekly sessions logged to 16 out of 18 (89%) logged. The average call length was 51 min and ranged from 33 to 68 min across the six mentee–peer mentor pairs. *Retention* All mentee–peer mentor pairs completed the intervention. Data collection at each time point was completed for all but one pair. Baseline data for one mentee and peer mentor pair were missing as researchers were not informed of their match by the peer support program coordinator until after weekly correspondence had commenced. Of the six mentees and six peer mentor participants in the intervention group, five mentees and five peer mentors participated in the qualitative component.	*Reinforcement* Participation in research was described by mentees and peer mentors as a way to give back and both mentees and peer mentors indicated that they may benefit “*in some way*” from the research. The monetary incentive was described as a “*bonus*” but was not cited as the primary factor for participation. Mentees indicated that they received benefits from the program (e.g., social connection) which reinforced their participation. Other described improvements (e.g., increased energy and motivation) also reinforced mentees’ participation in the program. *Environmental context and resources* Most mentees and peer mentors indicated that the researchers had provided an acceptable environment for conducting the research. The length of surveys and scheduling logistics were also deemed acceptable; however, there was a relatively high turnover of peer support program coordinators. Mentees and peer mentors appreciated the ability to determine program frequency according to their needs (e.g., adhering to a biweekly rather than weekly schedule), including the ability to skip calls for holidays and/or vacations. Mentees and peer mentors also highly appreciated the flexibility offered in scheduling outcome measurement given that factors such as their energy and focus changed from day to day and affected both their ability to participate, as well as their responses to survey items. This latter phenomenon was concerning to one mentee who questioned whether the research was capturing outcomes accurately.	*Some complementarity and some contradiction between quantitative and qualitative findings* Recruitment challenges reduced the study’s sample size and resultant power for meaningful statistical comparisons (despite the numerous benefits of partaking in research and adequate environmental context cited by mentees and peer mentors). A high turnover of peer support program coordinators may have also impacted adherence as peer support coordinators were responsible for communicating concerns or making changes (i.e., rescheduling) when required and hence had an impact on participants’ experiences; however, in almost all cases, mentee–peer mentor pairs exchanged information to facilitate this process for themselves. Therefore, interruptions to scheduling due to factors such as holidays or vacations may have been a more significant issue with regard to intervention adherence. While the program was not delivered once per week for all participants (i.e., some mentees and peer mentors agreed to modify the intervention to meet less frequently), responsiveness to contextual factors (e.g., energy and memory) as well as flexibility conceivably served to promote adherence (i.e., according to the wishes of mentees and peer mentors), as well as increase retention.
Community integration	*No statistically significant change* CIQ: overall mean score decreased from 16.1 (2.49) to 14.3 (6.65) for the intervention group and increased from 15.9 (4.88) to 17 (2.58) for the control group after four months (*g* = 0.58; CI = −0.591–1.751)	*Knowledge* Mentees and peer mentors both described the acquisition of knowledge as an outcome, including receiving and sharing knowledge of resources and tips for medications, mindfulness, and other everyday issues affected by brain injury (e.g., managing children). This also included reminders of existing knowledge, such as accessing brain injury resources or taking vitamins. *Skills* Mentees and peer mentors both experienced skill development due to the sharing of knowledge on multiple issues, including organization (e.g., using calendars and calendar reminders) and returning to work. *Goals* Both mentees and peer mentors described goals and goal setting as components of the program. Short-term goals were discussed. Peer mentors helped mentees with setting small, attainable goals which contributed to a larger, overall goal. One peer mentor also described realizing his own shortcomings in being more involved in the community and volunteering through conversations with his mentee and set his own goals to be more involved. *Social influences* Mentees and peer mentors both reported social support as an outcome; social support arose as the most dominant theme of the interviews. Reinforcement and validation were reported through the mutual sharing of similar experiences. Social support was a topic of conversation (i.e., discussion around family and romantic relationships).	*Contradiction between quantitative and qualitative findings* For a relatively short-term study (i.e., data collection in the current study was constricted to a four-month timeframe compared to the regular OBIA Peer Support Program which can last up to a year), community integration may not be a feasible outcome to measure and/or improve for partners. Connecting to and volunteering within the community were both topics which were infrequently discussed by mentees. However, progress in other areas, including knowledge, skills, goals, and social support suggest that it is conceivable that the short-term benefits of peer support occur on an individual, rather than community level. While community integration may be too distal an outcome to achieve within a four-month timeline, several of the qualitative outcomes described by mentees correspond to subscales of the CIQ and may represent specific mechanisms by which community integration can occur. Hearing about a peer mentor’s successes in raising children, returning to work, or maintaining relationships with family and friends may inspire increased awareness and confidence and elicit individual benefits for mentees in specific domains.
Mood	*No statistically significant change* PHQ-9: mean score increased from 14.2 (7.25) to 18.8 (8.96) for the intervention group and from 13.3 (7.09) to 13.7 (8.96) for the control group after two months. Mean score decreased to 10.4 (5.72) for the intervention group and to 13.0 (5.89) for the control group after four months (*g* = 0.447; CI = −0.715–1.608) Note: a higher PHQ-9 score is indicative of a higher level of depression severity	*Optimism* Mentees identified optimism as an outcome or intended outcome of the program. Mentees derived hope and inspiration from their mentors for progressing through their recovery. *Emotion* Positive and negative emotions were experienced by both mentees and peer mentors. Positive affect was described as generally feeling better after speaking with someone. Negative affect was described as an outcome for both partners and mentors if the topic of a conversation was particularly depressing or negative. Peer mentors experienced negative affect due to difficult conversations and emotional investment in their partner or feelings of unfulfillment due to an unsuitable match.	*Some complementarity and some contradiction between quantitative and qualitative findings* Recognizing the cyclical nature of mood (and of the interactions between mentees and peer mentors) is important to the synthesis of the mood-related findings. The nature of the discussions between mentees and peer mentors, particularly early on in the program, may have been a key factor in mediating the initial increases in depression severity for both the intervention and control groups after two months, as well as the negative emotions experienced by mentees. Discussion of more burdensome topics and/or difficult issues (e.g., the brain injury itself) at the start of the program may be expected due to mentees’ and peer mentors’ desires to address acute or critical issues as soon as possible (i.e., before broadening the conversations to other topics). While such conversations can serve an exploratory role and help form a strong connection early on so that mentees and peer mentors can easily build rapport, they may also elicit negative emotions in some cases (e.g., due to compassion fatigue by peer mentors). It is conceivable that as discussions progress to other topics of interest, such as hobbies or relationships, conversations occurring later in the program may ultimately elicit more positive emotions. Further exploration of this trend (i.e., after four months) is warranted given the individual variances which can occur in mood and the small sample size used in our study. The cyclical nature of mood also underscores the importance of finding appropriate mentee–peer mentor matches so that conversations continue to remain engaging and deliver hope and inspiration to mentees, thereby promoting positive emotions for the duration of the program.
Health-related quality of life	SF-20: statistically significant lower mean “pain” health concept score for the intervention group compared to the control group at two months (*p* = 0.021; *g* = 0.187; CI = −0.915–1.28) SF-20: statistically significant increase in mean “role functioning” health concept score for the intervention and control groups after four months (*p* = 0.05), but no difference between groups (i.e., interaction) was observed No other statistically significant changes	*Knowledge* Mentees and peeer mentors received and shared tips on strategies around symptom management (e.g., memory, organization), medications, and pain management. *Skills* Mentees and peer mentors both experienced skill development due to the sharing of knowledge on multiple issues, including organization (e.g., using calendars and calendar reminders) and returning to work. *Emotion* Positive and negative emotions were experienced by both mentees and peer mentors. Positive affect was described as generally feeling better after speaking with someone. Negative affect was described as an outcome for both mentees and peer mentors if the topic of a conversation was particularly depressing or negative. *Beliefs about capabilities* Mentees felt more confident in their decisions and the ways they have done things due to validation from peer mentors. Mentees and peer mentors experienced greater perceived competence and self-efficacy and felt more empowered.	*Some complementarity between quantitative and qualitative findings* Peer support programs may lead to improvements in pain via the sharing of knowledge on pain management techniques. Focused discussions on concrete strategies to employ for pain management (e.g., use of physical aids, medications, and/or meditation) may help develop mentees’ awareness and contribute to the exploration and use of additional, potentially helpful approaches by mentees. While SF-20 scores did not increase after four months for all health concepts, it is conceivable from our qualitative results that improvements in knowledge, skills, positive emotion, and beliefs about capabilities may all act as mechanisms of action for enhancing health-related quality of life. Discussions on daily activities and/or returning to work may have served to improve knowledge and skills to help with role functioning; however, since the control group also experienced a significant increase in role functioning after four months, it is conceivable that this health concept is one which naturally improves over time in patients as part of typical recovery progression. Beliefs about capabilities may also act as mediators to some SF-20 health concepts depending on factors such as a mentee’s individual emotions or mindset during peer support.
Self-efficacy	*No statistically significant change* TBI-SE: total mean score decreased from 75 (38.18) to 68.7 (28.55) for the intervention group and increased from 82.1 (30.73) to 83.3 (21.55) for the control group after four months (*g* = 0.585; CI = −0.529–1.698) TBI-SE: greater decrease in mean “social and community” subscale score for the intervention group compared to the control group after four months (trend toward significance; *p* = 0.063)	*Knowledge* Peer mentors shared knowledge of approaches or strategies that have previously worked for them (e.g., resource knowledge sharing) but respected professional boundaries by limiting their provision of specific advice to mentees. *Skills* Various skills (e.g., dealing with exhaustion and memory issues) were discussed as topics of conversation. Development of interpersonal skills was described as an outcome of participating in peer support by both mentees and peer mentors. Socializing through the program led to enhanced phone skills, including increased comfort and ease of speaking with someone else. Peer mentors reported improvements in mentor-related skills, including listening, selflessness, and having difficult conversations. *Beliefs about capabilities* Self-confidence, perceived competence, self-efficacy, and empowerment were experienced by both mentees and peer mentors. Mentees reported feeling more confident in their actions and decisions following validation from mentors. Improved self-esteem was also noted as an outcome by one mentee. *Reinforcement* Reinforcement was used as a strategy by peer mentors during conversations for partners’ actions or for the goals which mentees had set.	*Contradiction between quantitative and qualitative findings* While TBI-SE scores decreased for the intervention group after four months, our qualitative findings indicate that vicarious experience may help to promote mentees’ knowledge and skills, which may both serve as vehicles for ultimate improvement of self-efficacy. However, although hearing peer mentors’ success stories is conceivably advantageous and encouraging for mentees, it is also possible that such discussions may be overwhelming or even demotivating in some cases. Mentees may feel discouraged if discussions reveal significant gaps between their current situation/progression in recovery, compared to that of their peer mentor. This situation may be especially pertinent for mentees in an early stage of recovery or at the beginning of the peer support program. It is conceivable that mentees’ receptions to such discussions and consequent changes in self-efficacy may be mediated in part by factors such as emotions or mood (e.g., if a mentee feels optimistic or pessimistic while discussing his or her peer mentor’s successes).

CIQ: Community Integration Questionnaire; OBIA: Ontario Brain Injury Association; PHQ: Patient Health Questionnaire-9; SF-20: Medical Outcomes Study Short Form-20; TBI-SE: TBI Self-Efficacy Questionnaire.

## Data Availability

All data generated or analyzed during this study are included in this published article (and its Supplementary Materials information files).

## Supplementary Materials

The following are available online at https://www.mdpi.com/article/10.3390/jcm10132913/s1, Table S1. Community integration results at baseline, two months, and intervention completion; Table S2. Mood results at baseline, two months, and intervention completion; Table S3. Health-related quality of life results at baseline, two months, and intervention completion; Table S4. Self-efficacy results at baseline, two months, and intervention completion.
