# Supplementary material for: A Pilot Feasibility Randomized Controlled Trial on the Ontario Brain Injury Association Peer Support Program
Source: J Clin Med. 2021 Jun 29;10(13):2913. doi: 10.3390/jcm10132913 (PMC8269307; doi:10.3390/jcm10132913)
Supplement: Supplementary file 1 [file jcm-10-02913-s001.zip › jcm-1280047 XML Supplementary.pdf]

Table S1. Community integration results at baseline, two months, and intervention completion.

**Outcome Measure**	**Baseline**			**2 Months**			**4 Months**				
**CIQ Home Integration**	**a, n=4**	**b, n=7**	***p*-value**	**a, n=5**	**b, n=7**	***p*-value**	a, n=5	b, n=7	*p*-value	Hedge's *g* (95% CI)*	d_{ppc}
Mean (SD)	4.6 (2.31)	5.8 (2.75)	0.51	5.3 (2.40)	5.3 (1.87)	0.977	3.6 (2.67)	5.4 (2.15)	0.217	0.759 (-0.428 - 1.946)	-0.214
Median (IQR)	5.5 (4)	5 (5)	1	6.3 (3.75)	5 (2.5)	0.558	3 (5)	5 (0)	1		
CIQ Social Integration	a, n=5	b, n=7	*p*-value	a, n=5	b, n=7	*p*-value	a, n=4	b, n=7	*p*-value	Hedge's *g* (95% CI)*	d_{ppc}
Mean (SD)	7 (3.16)	7 (2.65)	1	7 (2.91)	7.6 (2.64)	0.73	7.5 (3.11)	8.4 (1.27)	0.602	0.434 (-0.808 - 1.676)	-0.29
Median (IQR)	8 (5.5)	7 (4)	0.876	8 (5.5)	7 (4)	0.755	7.5 (6)	8(3)	0.648		
CIQ Productivity	a, n=5	b, n=7	*p*-value	a, n=6	b, n=7	*p*-value	a, n=6	b, n=7	*p*-value	Hedge's *g* (95% CI)*	d_{ppc}
Mean (SD)	2.6 (1.95)	3.1 (1.77)	0.626	2.5 (1.52)	2.9 (2.11)	0.738	2.7 (1.75)	3.1 (2.04)	0.663	0.209 (-0.884 - 1.302)	0.05
Median (IQR)	2 (3.5)	3 (3)	0.639	2.5(2.5)	3 (4)	0.836	2.5(2.75)	2(4)	0.836		
CIQ Total	a, n=4	b, n=7	*p*-value	a, n=5	b, n=7	*p*-value	a, n=4	b, n=7	*p*-value	Hedge's *g* (95% CI)*	d_{ppc}
Mean (SD)	16.1 (2.49)	15.9 (4.88)	0.932	15.3 (4.83)	15.7 (3.29)	0.846	14.3 (6.65)	17 (2.58)	0.288	0.617 (-0.639 - 1.872)	-0.654
Median (IQR)	15.6 (4.62)	15 (5.25)	1	17.3(9.25)	14 (4.5)	0.876	12.1 (9.75)	16 (5)	0.23		

*A Hedge's g of 0.2, 0.5 and 0.8 represented a small, moderate and large effect size, respectively (Lakens, 2013). These same cut points were used to interpret d_{ppc}.

Table S2. Mood results at baseline, two months, and intervention completion.

	Baseline			2 Months			4 Months				
**PHQ-9**	**a, n=5**	**b, n=7**	***p*-value**	**a, n=5**	**b, n=6**	***p*-value**	**a, n=5**	b, n=7	*p*-value	Hedge's *g* (95% CI)*	d_{ppc}
Mean (SD)	14.2 (7.25)	13.3 (7.09)	0.832	18.8 (8.96)	13.7 (8.69)	0.347	10.4 (5.72)	13.0 (5.89)	0.463	0.447 (-0.715 - 1.608)	-0.452
Median (IQR)	13 (13)	17 (12)	1	15 (16.5)	15 (12)	0.53	10 (11)	15 (11)	0.432		
PHQ-9 Groups	a, n=4	b, n=7	*p*-value	a, n=4	b, n=7	*p*-value	a, n=5	b, n=7	*p*-value		
None/minimal	0 (0.0)	1 (14.3)	0.391	0 (0.0)	1(14.3)	0.608	1 (16.7)	1 (14.3)	0.926		
Mild	1 (16.7)	3 (28.6)		1 (16.7)	1(14.3)		1 (16.7)	1 (14.3)			
Moderate	2 (33.3)	0 (0.0)		1 (16.7)	1(14.3)		1 (16.7)	1 (14.3)			
Moderately severe	1 (16.7)	3 (42.9)		1 (16.7)	3(42.9)		2 (33.3)	0 (0.0)			
Severe	1 (16.7)	1 (14.3)		0 (0.0)	0 (0.0)		0 (0.0)	1 (14.3)			
Missing	1 (16.7)	0 (0.0)		2 (33.3)	1 (14.3)		0 (0.0)	0 (0.0)			

***A Hedge's g of 0.2**, 0.5 and 0.8 represented a small, moderate and large effect size, respectively (Lakens, 2013).

Table S3. Health-related quality of life results at baseline, two months, and intervention completion.

**SF-20**	**Baseline**			**2 Months**			**4 Months**				
**Physical Functioning**	**a, n=5**	**b, n=7**	***p*-value**	**a, n=6**	**b, n=7**	***p*-value**	a, n=6	b, n=7	*p*-value	Hedge's *g* (95% CI)*	d_{ppc}
Mean (SD)	65 (40.99)	52.4 (30.70)	0.554	51.4(48.42)	48.8 (30.21)	0.909	59.7 (47.26)	45.2 (35.96)	0.543	-0.35 (-1.448 - 0.749)	0.05
Median (IQR)	83.3 (70.83)	50 (42.3)	0.53	54.2 (100)	33.3 (58.33)	1	79.2 (100)	41.7 (66.6)	0.628		

Role Functioning	a, n=5	b, n=7	*p*-value	a, n=6	b, n=7	*p*-value	a, n=6	b, n=7	*p*-value	Hedge's *g* (95% CI)*	d_{ppc}
Mean (SD)	20 (44.72)	28.6 (48.80)	0.763	25 (41.83)	28.6 (39.34)	0.877	54.2 (45.87)	42.9 (47.24)	0.671	-0.242 (-1.337 - 0.852)	0.38 9
Median (IQR)	0 (50)	0 (100)	0.876	0 (62.5)	0 (50)	0.836	62.5 (100)	25 (100)	0.731		

Social Functioning	a, n=5	b, n=7	*p*-value	a, n=6	b, n=7	*p*-value	a, n=6	b, n=7	*p*-value	Hedge's *g* (95% CI)*	d_{ppc}
Mean (SD)	60 (40.0)	57.1 (42.31)	0.909	46.7 (41.31)	48.6 (39.76)	0.934	33.3 (37.24)	60 (38.30)	0.231	0.706 (-0.418 - 1.83)	-0.66
Median (IQR)	80 (70)	80 (80)	1	50 (85)	40 (80)	0.945	30 (65)	60 (80)	0.234		

Mental Health	a, n=5	b, n=7	*p*-value	a, n=6	b, n=7	*p*-value	a, n=6	b, n=7	*p*-value	Hedge's *g* (95% CI)*	d_{ppc}
Mean (SD)	50.4 (21.28)	56.0(13.27)	0.585	47.3 (20.77)	52 (13.06)	0.632	51.3 (20.15)	53.1 (12.59)	0.847	0.109 (-0.982 - 1.201)	0.102
Median (IQR)	56 (38)	60 (28)	0.639	48 (43)	52 (16)	0.731	52 (35)	56 (20)	0.945		

Health Perceptions	a, n=5	b, n=7	*p*-value	a, n=6	b, n=7	*p*-value	a, n=6	b, n=7	*p*-value	Hedge's *g* (95% CI)*	d_{ppc}
Mean (SD)	41 (35.60)	45 (26.61)	0.827	36.7 (30.27)	41.4 (27.49)	0.772	48.3 (31.09)	46.4 (22.12)	0.9	-0.071 (-1.162 - 1.019)	0.165
Median (IQR)	35 (70)	50 (40)	1	30 (62.5)	40 (45)	0.836	52.5 (49)	55 (35)	1		

Pain	a, n=5	b, n=7	*p*-value	a, n=6	b, n=7	*p*-value	a, n=6	b, n=7	*p*-value	Hedge's *g* (95% CI)*	d_{ppc}
Mean (SD)	36 (40.99)	60 (23.09)	0.222	36.7 (23.38)	68.6 (19.52)	***0.021***	60 (40)	65.7 (19.02)	0.758	0.187 (-0.905 - 1.28)	0.536
Median (IQR)	40 (70)	60 (40)	0.202	40 (45)	60 (20)	***0.035***	60 (70)	60 (20)	0.836		

*A Hedge's g of 0.2, 0.5 and 0.8 represented a small, moderate and large effect size, respectively (Lakens, 2013).

Bold with italics indicates statistical significance ($p<0.05$).

Table S4. Self-efficacy results at baseline, two months, and intervention completion.

Self-efficacy	Baseline			2 Months			4 Months				
**Social and community**	**a, n=5**	**b, n=7**	***p*-value**	**a, n=6**	**b, n=7**	*p*-value	a, n=6	b, n=7	*p*-value	Hedge's *g* (95% CI)*	d_{ppc}
Mean (SD)	30.4 (9.18)	29.1 (7.22)	0.795	21.8 (11.75)	29 (8.79)	0.235	21.7 (11.55)	30.4 (5.13)	0.132	1.005 (-0.152 - 2.161)	-1.145
Median (IQR)	32 (17)	29 (14)	0.755	24 (22)	29 (17)	0.366	25 (21)	29 (7)	0.366		
Physical functioning	a, n=5	b, n=7	*p*-value	a, n=5	b, n=7	*p*-value	a, n=6	b, n=7	*p*-value	Hedge's *g* (95% CI)*	d_{ppc}
Mean (SD)	5.2 (3.49)	6.1 (2.67)	0.606	4.3 (3.20)	6.6 (2.51)	0.185	6 (2.76)	6.9 (2.19)	0.545	0.365 (-0.734 - 1.464)	0
Median (IQR)	5 (6.5)	7 (4)	0.639	4 (7)	5 (5)	0.234	7 (3.5)	8 (3)	0.628		
Cognitive functioning	a, n=5	b, n=7	*p*-value	a, n=6	b, n=7	*p*-value	a, n=6	b, n=7	*p*-value	Hedge's *g* (95% CI)*	d_{ppc}
Mean (SD)	17.6 (11.65)	23 (11.46)	0.443	15 (8.44)	21.6 (9.95)	0.23	19.7 (9.93)	25 (10.61)	0.372	0.514 (-0.594 - 1.622)	0.008
Median (IQR)	21 (22.5)	24 (21)	0.53	15 (13)	20 (17)	0.366	20 (20.5)	26 (18)	0.295		
Emotional regulation	a, n=5	b, n=7	*p*-value	a, n=5	b, n=7	*p*-value	a, n=6	b, n=7	*p*-value	Hedge's *g* (95% CI)*	d_{ppc}
Mean (SD)	21.8 (15.16)	23.9 (11.36)	0.793	17.7 (11.08)	21.6 (9.71)	0.512	21.3 (9.95)	21 (8.41)	0.949	-0.033 (-1.123 - 1.058	0.17
Median (IQR)	29 (29)	27 (22)	0.876	18.5 (20.25)	20 (8)	0.836	22.5 (15.75)	22 (15)	0.945		
Total	a, n=5	b, n=7	*p*-value	a, n=5	b, n=7	*p*-value	a, n=6	b, n=7	*p*-value	Hedge's *g* (95% CI)*	d_{ppc}
Mean (SD)	75 (38.18)	82.1 (30.73)	0.727	58.8 (33.30)	78.7 (25.22)	0.246	68.7 (28.55)	83.3 (21.55)	0.315	0.585 (-0.529 - 1.698)	-0.204
Median (IQR)	87 (75)	85 (52)	0.755	61 (67.25)	74 (30)	0.534	75.5 (43.25)	83 (40)	0.628		

*A Hedge's g of 0.2, 0.5 and 0.8 represented a small, moderate and large effect size, respectively (Lakens, 2013).
